# Asthma Cure

**Published:** 1880-12

**Authors:** 


### ASTHMA CURE.

Belladonna leaves, two ounces ; stramonium leaves, one ounce ; powdered nitrate of potassium, one and one-half drachms. Mix them thoroughly. To be used by igniting a drachm in a suitable vessel and inhaling the fumes.

N. B.—A better plan is to go to Texas and remain there.—Ed.

### ROSE SACHET.

Rose leaves, ground, four ounces ; sandalwood, ground, two ounces ; oil of rose, one drachm ; powdered orris root, one ounce. Mix them.

"Two men got into a fight in front of the bank to-day," said a stock-broker at the family supper-table, "and it looked pretty hard for one of them. The biggest one grabbed a cart-stake and drew it back. I felt that he was going to knock the other's brains out, and I jumped between them."

The family had listened with rapt attention, and, as the head paused in his narrative, the young heir, whose respect for his father's bravery was immeasurable, proudly remarked, "He couldn't knock any brains out of you, could he, father ?"

The head of the family gazed long and earnestly at the heir, as if to detect evidences of a dawning humorist ; but as the youth continued with great innocence to munch his tart, he gasped and resumed his supper.

